# Rapid differentiation of *Francisella *species and subspecies by fluorescent in situ hybridization targeting the 23S rRNA

**DOI:** 10.1186/1471-2180-10-72

**Published:** 2010-03-08

**Authors:** Wolf D Splettstoesser, Erik Seibold, Ella Zeman, Karlheinz Trebesius, Andreas Podbielski

**Affiliations:** 1 Bundeswehr Institute of Microbiology, German Reference Laboratory for Tularemia, Neuherbergstr 11, 80937 Munich, Germany; 2 University of Applied Sciences Munich, Lothstr. 34, 80335 Munich, Germany; 3 Department of Microbiology, Virology & Hygiene, University Hospital Rostock, Schillingallee 70, 18057 Rostock, Germany

## Abstract

**Background:**

*Francisella (F.) tularensis *is the causative agent of tularemia. Due to its low infectious dose, ease of dissemination and high case fatality rate, *F. tularensis *was the subject in diverse biological weapons programs and is among the top six agents with high potential if misused in bioterrorism. Microbiological diagnosis is cumbersome and time-consuming. Methods for the direct detection of the pathogen (immunofluorescence, PCR) have been developed but are restricted to reference laboratories.

**Results:**

The complete 23S rRNA genes of representative strains of *F. philomiragia *and all subspecies of *F. tularensis *were sequenced. Single nucleotide polymorphisms on species and subspecies level were confirmed by partial amplification and sequencing of 24 additional strains. Fluorescent In Situ Hybridization (FISH) assays were established using species- and subspecies-specific probes.

Different FISH protocols allowed the positive identification of all 4 *F. philomiragia *strains, and more than 40 *F. tularensis *strains tested. By combination of different probes, it was possible to differentiate the *F. tularensis *subspecies *holarctica, tularensis, mediasiatica *and *novicida*. No cross reactivity with strains of 71 clinically relevant bacterial species was observed. FISH was also successfully applied to detect different *F. tularensis *strains in infected cells or tissue samples. In blood culture systems spiked with *F. tularensis*, bacterial cells of different subspecies could be separated within single samples.

**Conclusion:**

We could show that FISH targeting the 23S rRNA gene is a rapid and versatile method for the identification and differentiation of *F. tularensis *isolates from both laboratory cultures and clinical samples.

## Background

Tularemia is a zoonotic disease caused by the highly infectious, virulent, gram-negative bacterium *F. tularensis*. This bacterial disease occurs in various clinical forms depending on the route of inoculation and the virulence of the *F. tularensis *strain involved [1]. The geographical distribution of *F. tularensis *was long regarded to be restricted to the Northern Hemisphere [2], and only very recently *F. tularensis*-like strains have been cultured in Queensland, Australia [3], and Thailand, South-East Asia [4]. *F. tularensis *has a broad host range and can affect more animal species than any other zoonotic pathogen [2]. Whereas human infections in North America are mainly due to tick bites or contact with rabbits, several enzootic cycles have been described in the Eurasia. Here, *F. tularensis *is often associated with water and aquatic fauna and its transmission is considered to be more complex involving blood-sucking arthropods like mosquitoes or ticks or direct contact with infected mammals [5,6].

Due to its infectious nature, ease of dissemination and high case fatality rate especially in respiratory infection, *F. tularensis *was the subject in diverse military biological weapons programs and is still included among the top six agents with high potential to be misused in bioterrorism [7].

The taxonomic position of *F. tularensis *is complex and has changed frequently. At present, the *Francisellacae *family contains four validly published species: *F. tularensis, F. novicida, F. noatunensis *and *F. philomiragia. F. philomiragia *is an opportunistic pathogen which has been rarely isolated from immuno-compromised individuals [8]. *F. noatunensis *has been described to cause a granulomateous disease in fish [9,10]. *F. novicida *was shown to be very closely related to *F. tularensis*, and most scientific authors consider it to be the fourth subspecies (subsp.) of *F. tularensis *(*F. tularensis *subsp. *novicida*) [5,11]. In this paper we will follow this latter nomenclature. Very recently, two further *Francisella *species have been described [10,11]. Although the four subspecies of *F. tularensis *show close genetic and phenotypic relationship and have probably evolved from a common ancestor, they exhibit striking variation in virulence in humans and animals [1]. Only two subspecies cause the vast majority of clinical tularemia in mammals: *F. tularensis *subsp. *tularensis *(Type A), endemic in North America and *F. tularensis *subsp. *holarctia *(Type B) which is found in many countries of the holarctic region [5]. Both subspecies show different patterns in mortality and virulence in humans [12]. Type A isolates can cause a life-threatening infection whereas the less virulent type B isolates generally produce a milder disease. Strains of the subspecies *tularensis *can be further divided into two major clades, AI and AII, which seem to differ in virulence and to cause significant mortality differences in human infections [5,12]. In addition to the well known virulent strains classified into the subspecies described above, there are several lines of evidence showing that the genus *Francisella *may comprise additional, hitherto unknown species [13-15]. While some strains of *Francisella*-like bacteria had been grown from immuno-compromised patients [15,16], some putative *Francisella *species have been identified only by molecular means analyzing specimens from rodents, soil and water samples [13,15]. Moreover, similar uncultivable *Francisella*-like bacteria have been found in diverse tick species and are believed to represent endosymbionts of arthropods [17].

In clinical microbiology, the established cultivation and serological techniques are not sufficient for the diagnosis of all *Francisella *species or for a rapid and reliable discrimination of type A or type B tularemia. Cultivation of *F. tularensis *from clinical specimens requires at least two days; this is followed by detection of specific antigen, e.g. LPS and molecular typing. Some reports have identified unusual *F. tularensis *strains, isolated from patients or rodents, which lack cysteine requirement or production of regular *F. tularensis *LPS [15,16,18]. There is accumulating evidence, supported by recent molecular biological analyses, that *F. tularensis *may be difficult to recover in human and animal infection by using standard cultivation techniques, although direct immunofluorescence, immunohistochemical analysis or PCR allows detection of the organism within clinical samples [19-21].

Rapid identification of *F. tularensis *subspecies is important in the monitoring of enzootic tularemia during outbreaks of human tularemia or post-release scenarios [7,22,23].

Thus, rapid and reliable procedures for the direct detection and differentiation of *Francisellae *in clinical samples may prove helpful to both clinicians and public health authorities. Therefore, a 23S rRNA-based detection approach was developed, since this molecule has been used extensively to elucidate phylogenetic relationships of bacteria at intra- and intergeneric levels and it is also an excellent target for fluorescent in situ hybridization [24-26].

Near full-length 23S rRNA gene sequences for *F. philomiragia *and all four subspecies of *F. tularensis *were determined. Additional sequences for this target, which exists in three copies in the known *Francisella *genomes, were analyzed by extracting this information from the published whole genomes sequences currently available. These sequence data were used to develop additional primer sets and fluorescently labeled oligonucleotide probes suitable for species- and subspecies-specific fluorescent in situ hybridization (FISH) of pathogenic *Francisella *species in culture as well as clinical specimens.

## Methods

### Preparation of samples for in situ hybridization and PCR

All bacterial strains used in this study are listed in Table 1 and 2. *Francisella *strains were grown aerobically on heart cysteine agar (HCA) at 37°C and 5% CO_2_. All other strains were cultured on Columbia blood agar or in Luria-Bertani (LB) broth (BD, Heidelberg, Germany). Bacterial cells were harvested while in exponential phase, suspended in phosphate buffered saline (PBS), centrifuged, washed in PBS, resuspended in TE buffer (10 mM Tris, 1 mM EDTA [pH8]), and adjusted to an optical density of 1.0 at 600 nm. Bacterial suspensions were prepared for PCR analysis using the QIAGEN (Hilden, Germany) tissue kit as recommended by the manufacturer. For in situ hybridization, harvested cells were processed and fixed with paraformaldehyde (PFA) as previously described [27].

**Table 1:** Results of fluorescence in situ hybridization of all *Francisella (F.) tularensis *and *F. philomiragia *strains used in this study.

*Species and origin*	*Strain*	*Alt. designation*	*Hybridization probe*					
			**Bwall 1448**	**Bwphi 1448**	**Bwhol 1151**	**Bwnov 168**	**Bwtume 168II**	**Bwmed 1397**

***F. tul***. subsp. ***tularensis***								

Human, Ohio, 1941	FSC237	Schu S4	+	-	-	-	+	-

Squirrel, Georgia (USA)	FSC033	SnMF	+	-	-	-	+	-

Tick, BC, Canada, 1935	FSC041	Vavenby	+	-	-	-	+	-

Canada	FSC042	Utter	+	-	-	-	+	-

Hare Nevada, 1953	FSC054	Nevada 14	+	-	-	-	+	-

Human, Utah, 1920	FSC230	ATCC 6223	+	-	-	-	+	-

								

***F. tul***. subsp. ***holarctica***								

Live vaccine strain, Russia	F49	ATCC 29684	+	-	+	-	-	-

Origin unknown	F1	BGA	+	-	+	-	-	-

Human, Austria, 1997	F2		+	-	+	-	-	-

Human, Austria (Wien), 1997	F11		+	-	+	-	-	-

Hare, Austria, 1994 (4 isolates)	F6-7, F54-55		+	-	+	-	-	-

Hare, Austria, 1995	F8		+	-	+	-	-	-

Hare, Austria, 1996	F9		+	-	+	-	-	-

Hare, Austria, 1997 (13 isolates)	F12-F24		+	-	+	-	-	-

Hare, Austria, 1998 (2 isolates)	F46, F47		+	-	+	-	-	-

Macaque, Germany, 2005 (4 isolates)	F 101, F107-109		+	-	+	-	-	-

Water vole, Germany, 2005, (2 isolates)	F105-106		+	-	+	-	-	-

								

***F. tul***. subsp. ***mediasiatica***								

Miday gerbil, Kazakhstan, 1965	F63	FSC147	+	-	-	-	+	+

Tick, Central Asia, 1982	F64	FSC148	+	-	-	-	+	+

Hare, Central Asia, 1965	F65	FSC149	+	-	-	-	+	+

								

***F. tul***. subsp. ***novicida***								

Water, Utah, 1950	F48	ATCC 15482	+	-	-	+	-	-

Human, UK, 2003	F58	FSC595	+	-	-	+	-	-

Human, Texas, 1991	F59	FSC156	+	-	-	+	-	-

Human, Texas, 1995	F60	FSC159	+	-	-	+	-	-

Spain (*novicida*-like)	F62	FSC454	+	-	-	+	-	-

								

***F. philomiragia***								

Water, Utah	F50	ATCC 25016	+^1^	+	-	-	-	-

Muskrat, Utah, 1959	F51	ATCC 25015	+^1^	+	-	-	-	-

Water, Utah, 1960	F93	ATCC 25017	+^1^	+	-	-	-	-

Water, Utah, 1960	F94	ATCC 25018	+^1^	+	-	-	-	-

Hybridization with specific probes was only performed if prepared cells had shown a positive signal with probe EUB388 (positive control) and no staining with probe NON-EUB388 (negative control).

+: positive signal; - negative signal with 6-FAM or Cy3-labeld probes

+^1^: Depending on the formamide concentration used for hybridization. For details refer to Results section.n

**Table 2:** Non-*Francisella *organisms from the strain collection of the Bundeswehr Institute of Microbiology tested to assess the specificity of the FISH assay

Species	Strain designation	EUB388	NON-EUB338	Bwall1448
*Acinetobacter baumannii*	DSMZ 7324	**+**	-	-

*Acinetobacter calcoaceticus*	DSMZ 30006	**+**	-	-

*Achromobacter ruhlandii*	DSMZ 633	**+**	-	-

*Achromobacter xylosoxidans *subsp. *denitrificans*	DSMZ 30026	**+**	-	-

*Aeromonas hydrophila*	ATCC 7966	**+**	-	-

*Alcaligenes faecalis subsp. feacalis*	DSMZ 30030	**+**	-	-

*Bacillus cereus*	DSMZ 4222	**+**	-	-

*Bacillus mycoides*	DSMZ 2048	**+**	-	-

*Bacillus stearothermophilus*	DSMZ 5943	**+**	-	-

*Bacillus subtilis*	DSMZ 2109	**+**	-	-

*Burkholderia cepacia*	B46, clinical isolate (PI)	**+**	-	-

*Burkholderia thailandensis*	DSMZ 13276	**+**	-	-

*Candida albicans*	DSMZ 1386	**+**	-	-

*Citrobacter freundii*	DSMZ 30039	**+**	-	-

*Citrobacter koseri*	DSMZ 4595	**+**	-	-

*Chromobacterium violaceum*	LMG 1267	**+**	-	-

*Clostridium difficile*	DSMZ 1296	**+**	-	-

*Eikenella corrodens*	DSMZ 8340	**+**	-	-

*Enterobacter aerogenes*	DSMZ 12058	**+**	-	-

*Enterobacter cloacae*	ATCC 13047	**+**	-	-

*Enterococcus faecalis*	DSMZ 2570	**+**	-	-

*Escherichia coli*	ATCC 25922	**+**	-	-

*Hämophilus influenzae*	DSMZ 4690	**+**	-	-

*Kingella denitrificans*	DSMZ 10202	**+**	-	-

*Kingella kingae*	DSMZ 7536	**+**	-	-

*Klebsiella pneumoniae*	DSMZ 30104	**+**	-	-

*Legionella pneumophila*	DSMZ 1296	**+**	-	-

*Listeria monocytogenes*	DSMZ 12464	**+**	-	-

*Moraxella catarrhalis*	DSMZ 9143	**+**	-	-

*Morganella morganii*	DSMZ 6675	**+**	-	-

*Neisseria meningitidis*	DSMZ 10036	**+**	-	-

*Ochrobactrum anthropi*	DSMZ 7216	**+**	-	-

*Pasteurella multocida*	DSMZ 5281	**+**	-	-

*Plesiomonas shigelloides*	DSMZ 8224	**+**	-	-

*Propionibacterium acnes*	DSMZ 1897	**+**	-	-

*Proteus mirabilis*	DSMZ 4479	**+**	-	-

*Proteus vulgaris*	DSMZ 30118	**+**	-	-

*Pseudomonas aeruginosa*	DSMZ 11810	**+**	-	-

*Pseudomonas putida*	DSMZ 291	**+**	-	-

*Pseudomonas stutzeri*	NCTC 10450	**+**	-	-

*Psychrobacter phenylpyruvicus*	DSMZ 7000	**+**	-	-

*Rahnella aquatilis*	DSMZ 4549	**+**	-	-

*Ralstonia pickettii*	DSMZ 6297	**+**	-	-

*Salmonella typhimurium*	ATCC 13311	**+**	-	-

*Salmonella urbana*	HR	**+**	-	-

*Serratia marcescens*	DSMZ 30121	**+**	-	-

*Serratia proteomaculans*	DSMZ 4543	**+**	-	-

*Shigella flexneri*	DSMZ 4782	**+**	-	-

*Sphingomonas paucimobilis*	DSMZ 1098	**+**	-	-

*Staphylococcus aureus*	DSMZ 346	**+**	-	-

*Staphylococcus epidermidis*	DSMZ 1798	**+**	-	-

*Staphylococcus hämolyticus*	DSMZ 20228	**+**	-	-

*Stenotrophomonas maltophilia*	DSMZ 50170	**+**	-	-

*Streptococcus agalactiae*	DSMZ 2134	**+**	-	-

*Streptococcus mitis*	DSMZ 12643	**+**	-	-

*Streptococcus pneumoniae*	DSMZ 20566	**+**	-	-

*Streptococcus pyogenes*	DSMZ 20565	**+**	-	-

*Vibrio cholerae*	El Tor	**+**	-	-

*Vibrio parahaemolyticus*	DSMZ 10027	**+**	-	-

*Y. enterocolitica *subsp. *enterocolitica*	ATCC 9610	**+**	-	-

*Yersinia enterocolitica *subsp. *Palearctica*	DSMZ 13030	**+**	-	-

*Yersinia kristensenii*	ATCC 33638	**+**	-	-

*Yersinia pestis*	EV76	**+**	-	-

*Yersinia pseudotuberculosis*	ATCC 29833	**+**	-	-

*Yersinia ruckeri*	ATCC 29473	**+**	-	-

*Yersinia frederiksenii*	ATCC 33641	**+**	-	-

*Yersinia bercovieri*	ATCC 43970	**+**	-	-

*Yersinia rohdei*	ATCC 43380	**+**	-	-

*Yersinia mollaretii*	ATCC 43969	**+**	-	-

*Yersinia aldovae*	ATCC 35236	**+**	-	-

*Yersinia intermedia*	ATCC 29909	**+**	-	-

Abbreviations used in Table 2:

ATCC: American Type Culture Collection; DSMZ: German Type culture collection; HR: General Hospital of Regen; NCTC: National Collection of Type Cultures, London; PI: Pettenkofer Institute for Medical Microbiology, Munich; LMG: *Culture collection *of the "Laboratorium voor Microbiologie", University Gent

### PCR amplification, sequencing of 23S rRNA gene, and single nucleotide polymorphism (SNP) analysis

**Figure 1:**
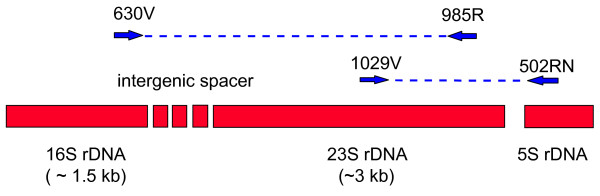
**Primer positions for the synthesis of two overlapping 23S rRNA gene fragments covering the complete 23S rRNA gene**. *Francisella tularensis *generally contains three rRNA operons in its entire genome. Analysis of the available whole genomes revealed that theses operons have identical nucleotide sequences.

Amplification and sequencing with universal primers of one strain of each *F. tularensis *subspecies as well as one strain of the species *F. philomiragia *were performed as described by Lane [28]. Full length amplification of 23S rDNA was obtained by combining primers which bind either to the 3'-end of the 16S rRNA gene and or the 5'-end oft 5S rRNA gene with primer sets specific for conserved regions within the 23S rDNA gene (Fig. 1, Additional file 1, Table S1). PCR reactions with these primer combinations were performed in a Hybaid thermocycler (MWG Biotech, Ebersberg, Germany) resulting in two complementary overlapping amplification products, which were purified (QIAGEN direct purification kit, QIAGEN, Hilden) and checked by gel-electrophoresis. Single-stranded DNAs were sequenced with multiple internal primers (Additional file 1, Table S1) using the LiCor system (MWG Biotech) and ThermoSequenase Cycle Sequencing kits (Amersham, Cleveland, USA). Sequences for both rRNA gene amplificates were determined, quality-checked and aligned. Single nucleotide polymorphisms specific for each subspecies or diverse combination of two subspecies were searched and are summarized in Additional file 1, Table S2.

### Development of PCR primers and hybridization probes

The alignment of all five complete 23S rRNA gene sequences and additional six sequences from publicly available whole *Francisella *genomes were used for the design of new PCR primers and hybridization probes. Primer and probe designations, sequences, positions, and references are listed in Additional file 1, Table S1. Three PCR protocols were developed in order to amplify variable 23S rRNA gene regions containing subspecies specific SNPs for 24 additional *Francisella *isolates comprising strains of each *F. tularensis *subspecies. For each PCR, 2 μL of DNA extracts were used. PCR was performed in a total volume of 50 μL containing 20 μL 5-Prime-MasterMix 2.5× (5 Prime, Hamburg, Germany) and 0.2 μM of each the forward and reverse primer. The PCR were performed with a GenAmp PCR System 9700 thermocyler (Applied Biosystems. Foster City, USA). Cycling conditions were: Initial denaturation at 94°C for 5 minutes, followed by 30 cycles of denaturation at 94°C for 40 seconds, annealing at 56°C for 30 seconds and amplification at 72°C for 90 seconds and a single final extension at 72°C for 5 minutes. The PCR products were purified with the QIAquick PCR Purification Kit™ according to the manufacturer's manual (Qiagen, Hilden, Germany).

The purified PCR products were sequenced with an BigDye^® ^Terminator v3.1 Cycle Sequencing Kit, Applied Biosystems (Applied Biosystems. Foster City, USA). The total volume of the sequencing reaction-mix was 10 μL containing 4 μL of the ready mix (BigDye^® ^Terminator v3.1 Cycle RR-100) from the kit, 3 μL of the purfied PCR product and 0.2 μM of the respective sequencing primer. Identical primers were used in both the amplification and sequencing PCR. All sequencing reactions were performed with the same thermocycler as described above. Cycler conditions were: An initial denaturation step at 96°C for 1 minute, followed by 25 cycles of denaturation at 96°C for 10 seconds, annealing at 50°C for 5 seconds, and extension at 60°C for 4 minutes. The sequencing products were purified with Centri-Sep spin columns (Princeton Separations, Adelphia, USA) and subsequently analyzed on an 3130 Genetic Analyzer (Applied Biosystems. Foster City, USA) in accordance with the instructions of the manufacturer.

Genus-, species- and subspecies-specific oligonucleotide probes for fluorescent insitu hybridization were developed using the software package ARB http://www.arb-home.de and probeBase http://www.microbial-ecology.net/probebase, synthesized and tagged with 6-FAM or Cy3 fluorescence dyes (MWG, Ebersberg, Germany).

### Whole-cell in situ hybridization

In situ hybridization on glass slides was performed as described previously [27]. For the detection or identification of each *Francisella *species or subspecies, two probe combinations were hybridized simultaneously to the reference cells and to the clinical samples in most cases. While testing the specificity and sensitivity of the newly developed probes, each preparation of reference cells from all different bacterial strains were additionally probed with a generic eubacterial probe (EUB338) and a non-sense nucleotide probe (NonEUB338) to confirm accessibility of the target rRNA as well as to exclude unspecific labelling of bacterial cells or tissue due to preparation artefacts [29]. Probes Bwall1448 and Bwphi1448 were used together to detect all *Francisella *species and to discriminate between *F. philomiragia *and *F. tularensis*. The combination of probes Bwtume168II and Bwmed1397 was applied in order to identify and discriminate *F. tularensis *subsp. *tularensis *(type A) and *F. tularensis *subsp. *mediasiatica*. Isolates of the subspecies *F. tularensis holarctica *and *F. tularensis *subsp. *novicida *were identified using probes Bwhol1151 or Bwnov168, respectively.

The addition of 30, 35 or 50% formamide to the hybridization buffer resulted in specific hybridization of the oligonucleotides to their respective target organisms. To reduce the amount of toxic waste, formamide was not used in the washing steps following hybridization. As a substitute, the NaCl concentration was decreased in the washing buffer according to the formula of Lathe [30] to obtain the necessary stringency. Citifluor (Citifluor Ltd., London, United Kingdom) was used as a mounting medium on hybridized slides, and the slides were examined both with a Leica (Heerbrugg, Switzerland) TCS NT scanning confocal microscope equipped with a standard filter set and a conventional fluorescence microscope (Axiostar plus/Axio CAM MR, Zeiss, Jena Germany). For probe excitation, an argonkrypton laser (Leica) or a mercurium-spectral light was used. Three different fluorochromes (DAPI, 6-FAM and Cy3) could be detected simultaneously with three different photomultipliers utilizing the green (6-FAM), red (Cy3), and blue (DAPI) channels of the Leica Application Suite (Leica) or Axiovision 4.5 (Zeiss) software packages. For the tissue sections, optical sectioning (0.5 to 1.0 μm width) was performed to reveal the three-dimensional localization of the probe-conferred fluorescence within the samples. The standard software delivered by the manufacturers was used to further process the digitized images.

### Identification of different *F. tularensis *subspecies in clinical material and infected cell cultures

Aerobic BACTEC blood culture bottles (BD, Heidelberg, Germany) were spiked with live bacterial cells from different *F. tularensis *subspecies. Single cultures were started with inoculums of 10 to 1000 colony forming units (cfu) in 5 ml whole human blood. Additionally, cells from two different subspecies were mixed at ratios of 1:1, 1:10, 1:100, 1:1000 and then cultured under aerobic conditions until the BACTEC instrument reported bacterial growth. Aliquots of 10 to 40 μl were dropped onto glass slides, air-dried and then fixed with PFA as described above.

Two different cell lines, the human monocyte/macrophage lineage U937 and the mouse macrophage cell line J 774 were infected with *F. tularensis *subsp. *holarctica *and *F. tularensis *subsp. *novicida *at a multiplicity of infection (MOI) of 100, incubated for 120 minutes and then fixed with paraformaldehyde [31]. Paraffin-embedded organs (spleen and liver) samples were sectioned with a microtome, fixed on glass slides, deparaffinized with alcohol and then subjected to the standard fluorescent in situ hybridization protocol.

### Nucleotide accession numbers

The nearly complete *23S rRNA gene *sequences of *F. tularensis *subsp. *mediasiatica *Francisella Strain Collection (FSC) 147, *F. tularensis *subsp. *tularensis *Schu S4, *F. philomiragia *ATCC 25017, *F. tularensis *subsp. *holarc*tica ATCC 29684, and *F. tularensis *subsp. *novicida *ATCC 15482, have been deposited under accession numbers GU073995 to GU073998 and GU073986, respectively.

The partial *23S rRNA gene *sequences of 24 additional *Francisella *strains have been deposited under accession numbers GU073970 to GU073985, and GU073987 to GU073994.

## Results

### Sequence analysis of the 23S rRNA gene and phylogeny

The PCR primers 630V, 985R, 1029V and 502RN directed the synthesis of two overlapping 23S rRNA gene fragments, which covered the complete 23S rRNA gene (Fig. 1). Complete double-stranded sequences of these amplicons were determined for the five strains *F. tularensis *subsp. *tularensis *Schu S4, *F. tularensis *subsp. *holarc*tica ATCC 29684, *F. tularensis *subsp. *mediasiatica *FSC 147, *F. tularensis *subsp. *novicida *ATCC 15482, and *F. philomiragia *ATCC 25017. The 23S rRNA gene sequences of the *F. tularensis *subspecies exhibited very high levels of homology (99.4 to 99.9% identity). Between *F. tularensis *subsp. *tularensis *FSC 237 (Schu S4) and *F. tularensis *subsp. *holarctica *(LVS, ATCC 29684) 11 different single nucleotide substitutions were found. Differences between *F. tularensis *subsp. *novicida *(ATCC 15482) and the three other subspecies ranged from 10 to 19 single nucleotide substitutions.

We identified regions of intrageneric or intraspecies variability that allowed discriminating between the species *F. tularensis *and *F. philomiragia*. In contrast to former results on the corresponding 16S rRNA gene sequences [32], the 23S rDNA genes displayed several single nucleotide polymorphisms (SNPs), which allowed a definite discrimination of *Francisella *strains on the subspecies level and even confirmed the differentiation of type AI and type AII clades (Additional file 1, Table S2).

### PCR for confirmation of SNP

**Figure 2:**
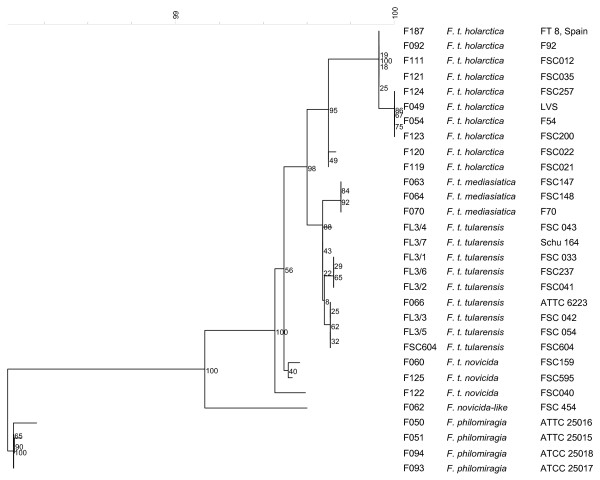
**Phylogentic tree based on nearly complete nucleotide sequences of *23S rRNA *gene of *F. tularensis *and *F. philomiragia *generated using a neighbor joining analysis**. Bootstrap values based on 1000 resamplings are indicated at branch points.

Three variable regions in the 23S rDNA genes were also sequenced in 24 additional *Francisella *strains using specific primers based on results from the initial sequence analysis. Thus, most of the SNPs shown in Additional file 1, Table S2 were confirmed. Only the single nucleotide substitution at position 913 (A versus G) was found to be specific for *F. tularensis *subsp. *tularensis *Schu S4 and other AI strains. All type AII strains displayed the *Francisella *consensus sequence at this position. The phylogenetic tree generated using a neighbor joining analysis procedure (Fig. 2) is consistent with data from the 16S rRNA gene and a whole genome SNP phylogeny [5] showing that *F. philomiragia *is clearly distal from the *F. tularensis *subspecies. Among the latter, strains of the subspecies *F. tularensis *subsp. *novicida *are distinct and derived from the ancestral lineage leading to the other subspecies. The phylogeny then separates into two lineages with *F. tularensis *subsp. *tularensis and F. tularensis *subsp. *mediasiatica *on one side and strains belonging to *F. tularensis *subsp. *holarctica *on the other. The type A strains can then be further separated into two groups corresponding to the known subtypes AI and AII.

### Sensitivity and specificity of in situ hybridization

**Figure 3:**
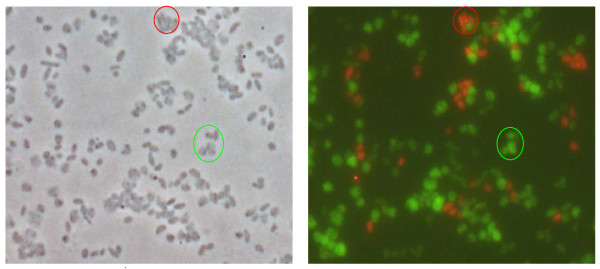
**Left: Artificial mixture of *F. tularensis *subsp. *tularensis *(Schu S4, red circle) and *F. philomiragia *(ATCC 25017, green circle), phase contrast microscopy**. Right: Fluorescence microscopy after hybridization with probes Bwall1448-Cy3 and Bwphi1448-6-FAM with 50% formamide. The green, pleomorphic cells of *F. philomiragia *can be easily distinguished from the smaller, coccoid rods of the highly virulent *F. tularensis *subsp. *tularensis *strain showing red fluorescence.

Probe Bwall1448 was targeted to an rRNA region unique for the genus *Francisella *and gave a positive signal for all *Francisella *strains tested in this study (Table 1). Due to a single mismatch within the binding site of this probe in *F. philomiragia *strains, the simultaneous application of a second probe (Bwphi1448), labeled with a different fluorochrome allowed the identification of all investigated *Francisella *strains on the species level by a single analysis (Fig. 3). Alternatively, each probe could be combined with EUB338, a probe targeted to a 16S rRNA-sequence conserved in most bacterial species, to rapidly exclude the presence of either *F. tularensis *or *F. philomiragia *in a bacteria-containing sample.

All five available *F. tularensis *subsp. *novicida *strains hybridized to the probes Bwall1448 as well as the *novicida*-specific probe Bwnov168 that binds within helix 10b of the 23S rRNA. Probe Bwhol1151 (binding site nt 1151 to nt 1170, helix 45) is complementary to all *F. tularensis holarctica *sequences and gave strong signals when hybridized to its respective target strains (Table 1). No cross reactivity with *F. tularensis *subsp. *tularensis *or other subspecies were observed.

**Figure 4:**
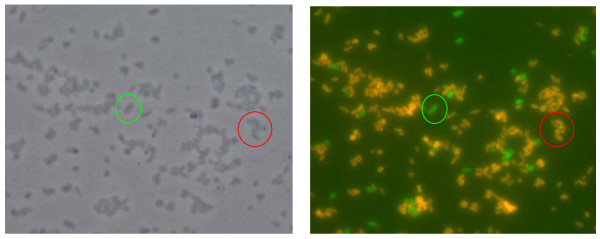
**Left: Artificial mixture of *F. tularensis *subsp. *tularensis *(Schu S4, green circle) and *F. tularensis *subsp. *mediasiatica *(FSC 148, red circle), phase contrast microscopy**. Right: Fluorescence microscopy after hybridization with probes Bwmed1379-Cy3 and Bwtume168II-6-FAM with 20% formamide. *F. tularensis *subsp. *tularensis *cells only bind to probe Bwtume168II-6-FAM (green fluorescence) whereas bacterial cells of *F. tularensis *subsp. *mediasiatica *bind to both probes resulting in a yellow-orange fluorescence.

**Figure 5:**
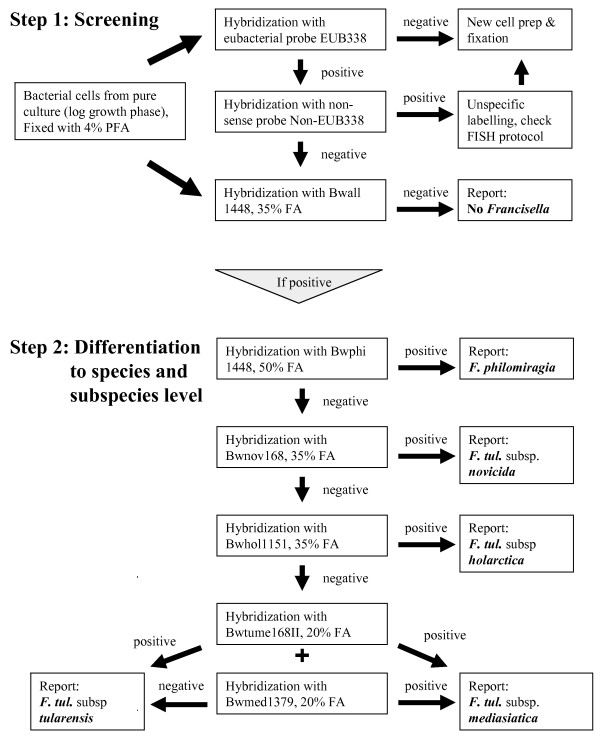
**Two-step algorithm for the rapid identification and differentiation of *Francisella *strains using fluorescence in situ hybridization**. After an initial hybridization step with three probes including the "pan-*Francisella*" probe Bw-all1488, negative samples can directly be reported. Performing internal controls with probe EUB-338 allows recognizing false negative results caused by technical problems. After hybridization with all species- and subspecies-specific probes in parallel, initially positive samples can be further differentiated by following the algorithm depicted in step two allowing unambiguous identification to subspecies level.

The lack of suitable SNPs specific for all *F. tularensis *subsp. *tularensis *strains made it necessary to develop a double-staining approach using two probes which are either complementary to all subspecies *mediasiatica *and *tularensis sequences (probe Bwtume168II) or only to the 23S rRNA of F. tularensis subsp.mediasiatica *(Bwmed1379) (Fig. 4). The first probe was directed to position nt 168 to 184 (helix 10b) which contains two SNPs which prevents its hybridization to sequences of *F. philomiragia*, *F. tularensis *subsp. *novicida *and type B strains. The second probe exclusively bound to the RNA of *F. tularensis *subsp. *mediaiasiatica *strains due to a single SNP located in the center of the probe binding site and discriminating these strains from all other gamma proteobacteria in the 23S rRNA database (Table 1). The simultaneous or consecutive application of all probes allows an unambiguous identification of a query isolate to the subspecies level within a few hours (Fig. 5).

### In situ detection and identification of *Francisella *bacterial cells in tissue samples, cell-, and blood-culture

**Figure 6:**
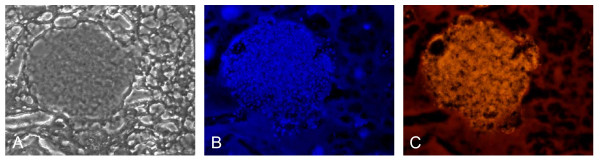
**Specific detection of *F. tularensis *subsp. *holarctica *in a liver tissue sample (mouse) fixed in formalin and embedded in paraffin for more than four years**. After deparaffinization and fluorescence in situ hybridization, bacterial cells can be visualized in small granuloma (A: phase contrast microscopy; B: fluorescence microscopy, DAPI staining; C: Specific staining of *F. tularensis *subsp. *holarctica*).

Spleen and liver paraffin sections from experimentally or naturally infected mice or non-human primates, were fixed, pre-treated to remove the embedding medium and then hybridized with probes EUB338, non-EUB338, Bwall1448, Bwnov168 and Bwhol1151. All tissue and cell culture samples showed moderate to strong autofluorescence. Despite such interference, the bacterial cells could be detected by using fluorescence microscopy and additional DNA staining with DAPI. In the infected tissue or cell culture samples, *F. tularensis *subsp. *holarctica *and *F. tularensis *subsp. *novicida *could then be identified by hybridization with their specific probes (Fig. 6 + 7).

**Figure 7:**
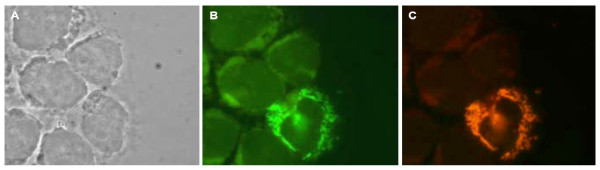
**Cytospin preparation of infected U 937 cell culture followed by specific detection of the facultative pathogen *F. tularensis *subsp. *novicida *(MOI 10:1, 24 h)**. (A: phase contrast microscopy; B: FISH, probe EUB338-6-FAM; C: FISH, probe Bwnov168-Cy3).

An automated blood culture system (BACTEC, BD, Heidelberg, Germany) was used to grow bacterial cells from each representative strain initially used for 23S rRNA gene sequencing. The culture bottles were spiked with 5 ml of human blood and the bacteria grown on HCA medium. Depending on the subspecies and the initial inoculum size, growth in aerobic blood culture bottles occurred between two to eleven days of incubation. Bacterial cells from each subspecies were strongly labeled with their corresponding probes as well as the EUB338 probe used for positive control (Table 3).

**Table 3:** Identification of different *F. philomiragia *and *F. tularensis *subspp. in positive blood culture using FISH.

	Bwall1448 (35% FA)	Bwphi1448 + Bwall1448_c_ (50%FA)	Bwhol1151 + Bwhol1151_c_ (35%FA)	Bwnov168 + Bwnov168_c_ (35%FA)	Bwtume168II + Bwtume168_c_ (20%FA)	Bwmed1379 + Bwmed1379_c_ (20%FA)
*F. tul*. subsp. *holarctica*	**+**	-	**+**	-	-	-

*F. tul*. subsp. *mediasiatica*	**+**	-	-	-	**+**	**+**

*F. tul*. subsp. *novicida*	**+**	-	-	**+**	-	-

*F. philomiragia*	**+**	**+**	-	-	-	-

Blood culture bottles were inoculated with 5 ml venous blood spiked with 10^2^CFU of each different strain.

+: positive hybridization

-: negative reaction, no fluorescence

**Figure 8:**
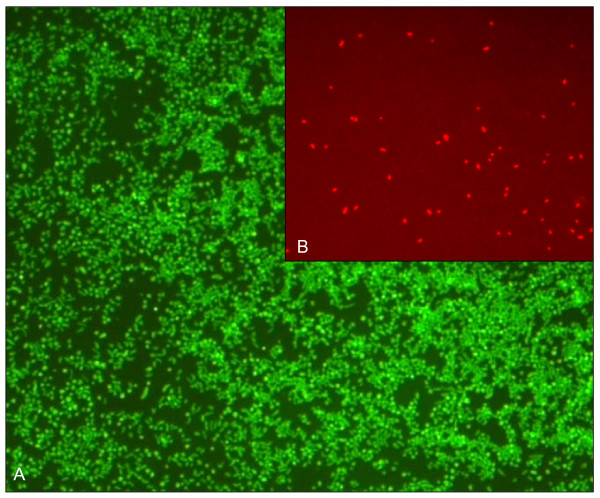
**Mixed sample of bacterial cells from *F. tularensis tularensis *(ATCC 6223) *and F. tularensis *subsp. *holarctica *LVS (ratio 100:1)**. Contamination lower than 1% could be identified using appropriate probe sets. (A: FISH staining with probe EUB338-6-FAM for staining of all bacteria in liquid samples. B: Specific staining of *F. tularensis *subsp. *holarctica*).

In mixed samples containing bacterial cells from different strains (e.g. type A as well as type B) both populations could be easily separated by whole cell hybridization with distinctly labeled probes (Fig. 8). By this approach, for instance, one type A bacterial cell can be detected and unequivocally identified in 1.000 type B cells.

## Discussion

Tularemia is a rare but dangerous zoonosis, which is endemic in almost all countries of the Northern Hemisphere. In some areas like Central and Southern Europe as well as Turkey, tularemia is an emerging or re-emerging disease representing a significant threat for public health [33-35]. Its causative agent, *F. tularensis*, is regarded as a potential biological warfare or bioterrorism agent of the highest category. For these reasons clinical and public health laboratories are urged to provide rapid and reliable diagnostic tools for the sensitive detection and identification of *F. tularensis *and for the appropriate differentiation of its subspecies or clades which often display significantly divergent pathogenic potential [12,22].

In contrast to most other bacterial pathogens, cultivation of *F. tularensis *is difficult due to its fastidious nature and its susceptibility to overgrowth by concomitant flora. Additionally, growth may be delayed (up to 12 days) and cultivation of *F. tularensis *poses a significant threat of laboratory infections. Only recently, conventional and real-time PCR protocols for the detection and identification of *F. tularensis *have been published, but still none of these techniques is sufficiently evaluated to be routinely used in clinical laboratories [36].

In this study we evaluated the potential of rRNA gene targeted PCR and sequencing as well as fluorescent in situ hybridization for the detection and differentiation of *Francisella *species. In- silico analysis of partial and complete 16S rRNA genes available in publicly accessible databases like GenBank confirmed the results of a previous study by showing that 16S rRNA sequences from *F. tularensis *subspecies are almost identical, and therefore, are only of limited value for the detection and discrimination of *F. tularensis *on the species or subspecies level [32]. In this regard, the difficulties to discriminate type A and type B strains resembled the situation in the closely related zoonotic pathogens *Yersinia (Y.) pseudotuberculosis *and *Y. pestis *or *Burkholderia (B.) pseudomallei *and *B. mallei *[25,37,38]

In contrast to those studies, comparison of full-length 23S rRNA genes of all *F. tularensis *subspecies as well as *F. philomiragia *revealed several discriminative SNPs. The sequence data obtained from rRNA gene sequences, known to be highly conserved in bacterial phylogeny, could be successfully used for the construction of hybridization probes, allowing a rapid genotype-based detection of *Francisella *species on different taxonomic levels.

A unique 23S rRNA target region suitable for the detection of *F. tularensis *subsp. *holarctica *(type B) could be identified. For the discrimination of *F. tularensis *subsp. *tularensis *(type A) and subsp. *mediasiatica*, an identification approach was developed by employing two different probes. Six type A strains, 31 type B strains as well as three *F. tularensis *subsp. *mediasiatica *strains were correctly identified by this approach, whereas no false-positive signal was observed with 71 other variably related bacterial species.

Similar results were gained employing species-specific probes for *F. philomiragia *and *F. tularensis*, which were tested with all mentioned *F. tularensis *strains as well as four *F. philomiragia strains*. We also developed an in situ hybridization protocol for *F. tularensis *subsp. *novicida*, which allowed the detection of all four available strains of this subspecies. However, due to the possible genetic variability within this subspecies [39] it remains to be elicited if the designed probe will also recognize other so called '*novicida*-like' strains [3,4,13-16]. While the final version of this manuscript was written, *23S rRNA *gene sequences of the aforementioned fish pathogenic members of the genus *Francisella *became publicly available [9,10]. An in silico analysis of these sequences revealed that strains of the species *F. noatunensis *will be probably detected by probe Bwall1448. The available data also indicate, that at it might be possible to discriminate between *F. noatunensis *comp. nov. and *F. noatunensis *subsp. *orientalis *if probe Bwphi1448 would be combined with probe Bwall1448. It is mandatory to experimentally verify these sequence-based predictions.

Caused by the genetic homogeneity and the clonal population structure of *F. tularensis*, discrimination of bacterial strains to the subspecies level by means of conventional PCR was almost impossible until 2003 [40]. Today, the application of different real-time PCR techniques using fluorescently labeled probes allows the discrimination of type A and type B strains from culture or clinical samples [20,41,42]. However, these techniques need sophisticated and expensive instrumentation and none of the published protocols are sufficiently validated to be directly used in routine microbiology.

Fluorescent oligonucleotide probing of whole cells is fast (less than two hours), reliable and could be analyzed by regular fluorescence microscopy, which is available in virtually all clinical or public health laboratories. In tularemia, immunofluorescence staining of clinical samples with anti-*F. tularensis *LPS antibodies is routinely applied [19], but antibodies discriminating the different subspecies are not available. Fluorescent in situ hybridization could be a rapid, complementary method to confirm preliminary results and to additionally allow the definitive identification of the respective subspecies that caused the infection. This could be important for the clinical patient management with respect to the known differences in type-specific virulence as well as for epidemiological investigations of tularemia outbreaks [23].

For two additional reasons, fluorescent in situ hybridization is a suitable alternative to biochemical identification or PCR. First, it can be applied to thoroughly inactivated clinical or culture samples thereby reducing the threat of laboratory infection. Second, it works without expensive and technical sophisticated devices, rendering FISH a cost-effective procedure. The potential for routine application of this method is supported by the availability of commercial test kits for clinically relevant species (e.g. *Pseudomonas aeruginosa*, *B. cepacia*) in typical patient specimens such as sputum or blood culture [24,43].

For the detection of *Y. pestis *and *Brucella *sp., other highly virulent bacterial species potentially misused as bioterrorism agents, similar protocols have successfully been developed [25,44].

The capability to detect and identify the closely related *Francisella *species within 2-3 hours without expensive preparation of nucleic acids from clinical samples is intriguing and may prove useful considering that misidentification of *F. tularensis *type A as *F. tularensis *type B and vice versa may occur when identification is based on the immunological detection of the LPS capsule or biochemical tests. In the past, such misidentification led to laboratory infections and resulted in the temporary shutdown of laboratories for cost-intensive decontamination [45]. The sensitivity of the new method is intriguing, since we were able to detect artificial contamination of type B strains with *F. tular*ensis type A as low as 0.1% of the total bacterial population. Moreover, FISH could prove relevant for the rapid identification of mutations in 16S or 23S rRNA gene regions which are associated with or causative for antibiotic resistance of bacterial pathogens against aminoglycosides or macrolides [46].

This investigation showed that *Francisella *cells infecting different mouse or primate tissues carry sufficient numbers of ribosomes to be detected with fluorochrome-labeled oligonucleotides. The probes readily penetrate tissue samples and bacterial cell walls. This technique is well suited to detect the location of a pathogen within the body, an advantage that can be further improved in combination with confocal laser scanning microscopy. This modification could compensate the comparatively low sensitivity of in situ hybridization typically requiring about 10^5 ^cells per ml for a positive reaction [25].

"Phylogenetic staining" using fluorescence labeled hybridization probes was employed for several clinically relevant and also environmental bacterial species [27]. For environmental studies, fluorescent in situ hybridization is used for the identification at genus, species and subspecies level especially for uncultivable species making FISH an extremely valuable tool to study ecological niches of bacterial species or symbiotic life styles in complex ecological systems. Future studies will show whether in situ hybridization techniques are sufficiently sensitive to detect dormant or metabolically inactive *Francisella *cells intracellularly surviving within tissues or in environmental samples like water, soil or arthropod vectors.

## Conclusions

The molecular methods investigated in this study offer alternatives to more traditional diagnostic methods for detection of tularemia in humans and animals. In particular, whole-cell hybridization is a promising, rapid, and cultivation-independent detection method for *Francisella*e in clinical samples but could also prove useful to detect and explore the newly recognized diversity of *Francisella *species or *Francisella*-like organisms in the environment.

## Authors' contributions

WDS conceived the study, participated in its design and coordination and drafted the manuscript. ES carried out the molecular genetic studies, analyzed the aligned sequences, constructed phylogenetic trees, participated in the study design and was involved in probe and primer design. EZ performed all hybridization experiments, 23S rRNA gene sequencing, and participated in sequence alignment, probe design and drafting the "methods" part of the manuscript. KT participated in study design, initial sequencing of the five reference strains, development of hybridization protocols and he was responsible for most of the probe design. AP contributed to study design and coordination, helped to draft the manuscript and critically revised its final version. All authors read and approved the final manuscript.

## Authors' informations

WDS and ES direct the German Reference Laboratory for Tularemia, which was repeatedly appointed by the Germany Federal Ministry of Health to provide specialist expertise in the field of tularemia. The predominant task is to give advice and support for special diagnostic problems. The reference laboratory supports physicians, clinical laboratories and public health institutions in diagnosis, treatment and surveillance of tularemia.

## Supplementary Material

Additional file 1 **Table S1 and S2**. Table S1: PCR primers and probes used in this study (Degenerate oligonucleotides wobble bases according to the IUB code). Table S2: Subspecies specific single nucleotide polymorphisms (SNPs) in the sequence of the 23S rRNA gene based on sequences of 29 *Francisella *strains. Click here for file
