# Supplementary material for: Rapid differentiation of Francisella species and subspecies by fluorescent in situ hybridization targeting the 23S rRNA
Source: BMC Microbiol. 2010 Mar 8;10:72. doi: 10.1186/1471-2180-10-72 (PMC2844405; doi:10.1186/1471-2180-10-72)
Supplement: Additional file 1 — Table S1 and S2. Table S1: PCR primers and probes used in this study (Degenerate oligonucleotides wobble bases according to the IUB code). Table S2: Subspecies specific single nucleotide polymorphisms (SNPs) in the sequence of the 23S rRNA gene based on sequences of 29 Francisella strains. [file 1471-2180-10-72-S1.DOC]

**Additional file 1, Table S1:** PCR primers and probes used in this study (Degenerate oligonucleotides wobble bases according to the IUB code)

| **Designation & Function** | **sequence 5' - 3'** | **Target/binding position** |
| --- | --- | --- |
| *Primers for complete 23S rRNA gene amplification* |  |  |
| 630V | GGA TCA CCT CCT TTM TG | 16S: 1529 - 1545 (*E.coli* sequence) |
| 1029V | CCG TAA CTT CGG GCG CCG G | 23S: 1685 – 1703 (*E.coli* sequence) |
| 985R | CCG GTC CTC TCG TAC T | 23S: 2654 – 2669 (*E.coli* sequence) |
| 502RN | TAT AGC GGC CGC SWG TTC GGR AWG GGA | 5S: 35 – 60 (*E.coli* sequence) |
|  |  |  |
| Pr*imers for complete 23SrRNA gene sequencing* |  |  |
| 1020R | TCT GGG YTG TTY CCC T | 23S: 975 – 909 (*E.coli* sequence) |
| 1025V | GGT AGG RGA GCD TTC | 23S: 1192 – 1206 (*E.coli* sequence) |
| 1037R | CGA CAA GGA ATT TCG CTA C | 23S: 1930 – 1948 (*E.coli* sequence) |
| 328V | TCC TAA GGT AGC GAA ATT CCT TG | 23S: 1928 – 1940 (*E.coli* sequence) |
| 1036V | ARA ACG TCG TGA GAC AG | 23S: 2587 – 2603 (*E.coli* sequence) |

**Additional file 1, Table S2:** Subspecies specific single nucleotide polymorphisms (SNPs) in the sequence of the 23S rRNA gene based on sequences of 29 *Francisella* strains. Assorted base substitutions in comparison to the species consensus sequence of all analysed *Francisella* strains are depicted. Positions (Pos.) refer to *E. c*o*li* K-12 23S rDNA sequence.

| ***Francisella tul****.* **subsp.** ***tularensis*** | ***Francisella tul.* subsp.** ***mediasiatica*** | ***Francisella tul.* subsp.** ***holarctica*** | ***Francisella tul.* subsp.** ***novicida*** |
| --- | --- | --- | --- |
|  |  |  | Pos. 152: G🡺A^a^ |
| Pos. 169: A🡺G ^b^ | Pos. 169: A🡺G ^b^ |  |  |
|  |  | Pos. 597: C🡺T |  |
| Pos. 913: G🡺A ^c^ |  |  |  |
|  |  | Pos. 1159: C🡺T ^e^ |  |
|  | Pos. 1389: A🡺G ^d^ |  |  |
|  | Pos. 2065: G🡺A |  |  |
|  |  | Pos. 2065: A🡺C |  |
|  |  | Pos. 2136: T🡺C |  |
| Pos. 2168: G🡺A ^f^ |  |  |  |
|  | Pos. 2727: A🡺G |  |  |
|  |  | Pos. 2736: C🡺T |  |

^a^ Used for probe Bwnov168

^b^ Specific for *F. tul.* subsp. *tularensis* and *F. tul.* subsp. *mediasiatica*. Used for probe Bwtume 168II

^c^ Specific for *F. tul*. subsp. *tularensis* Type AI

^d^ Used for probe Bwmed1379

^e^ Used for probe Bwhol1151

^f^ Specific for *F. tul.* subsp. *tularensis* Type AII
